# Multiplex PCR based genotypic characterization of pathogenic vancomycin resistant *Enterococcus faecalis* recovered from an Indian river along a city landscape

**DOI:** 10.1186/s40064-016-2870-5

**Published:** 2016-07-28

**Authors:** Pushpa Lata, Siya Ram, Rishi Shanker

**Affiliations:** 1 CSIR-Indian Institute of Toxicology Research, PO Box 80, MG Marg, Lucknow, U.P. 226001 India; 2 Institute of Life Sciences, School of Science and Technology, Ahmedabad University, University Road, Ahmedabad, 380009 India

**Keywords:** Mutiplex PCR, River water, Surface water, Vancomycin resistant *E. faecalis*

## Abstract

**Background:**

Enterococci are normal commensals of human gut, but vancomycin-resistant enterococci (VRE) are a severe threat to human health. Antimicrobial-resistant enterococci have been reported previously from Indian surface waters. However, the presence of antimicrobial resistance and virulence markers in *Enterococcus faecalis*, the most dominant enterococci is yet to be investigated.

**Objectives:**

The goal of this study was to analyse concentration of enterococci and distribution of antimicrobial resistance and virulence markers in *E. faecalis* isolates from river waters along an important north Indian city landscape.

**Methods:**

We enumerated enterococci in river water samples (n = 60) collected from five sites across the Lucknow city landscape using the most probable number and membrane-filtration methods. The antimicrobial sensitivity profile of *E. faecalis* isolate was generated with the Kirby-Bauer antimicrobial disc diffusion assay. The multiplex PCR was used for genotypic characterization of vancomycin-resistance and virulence in *E. faecalis* isolates.

**Results:**

Enterococci density (p < 0.0001) increased from up-to-down-stream sites. Multiplex PCR based genotypic characterization has shown a significant distribution of virulence-markers *gelE*, *ace* or *efaA* in the *E. faecalis* isolates (p < 0.05). The range of antimicrobial-resistance varied from 5 to 12 in the landscape with the frequency of vancomycin-resistant *E. faecalis* (VRE) ranging from 22 to 100 %.

**Conclusion:**

The occurrence of pathogenic VRE in river Gomti surface water is an important health concern. The observed high background pool of resistance and virulence in *E. faecalis* in river waters has the potential to disseminate more alarming antimicrobial resistance in the environment and poses serious health risk in developing countries like India as VRE infections could lead to increased cost of healthcare.

**Electronic supplementary material:**

The online version of this article (doi:10.1186/s40064-016-2870-5) contains supplementary material, which is available to authorized users.

## Background

Enterococci is considered to be a suitable ‘indicator’ of fecal contamination in aquatic environment as it survives longer compared to other fecal streptococci and coliforms (Cabelli et al. 1982; USEPA 2003). *Enterococcus faecalis*, most prevalent enterococci, is an enteric bacterium of mammalian intestinal tract. It is notorious as an opportunistic pathogen causing various infections (Thurlow et al. 2010; Parsek and Singh 2003). Majority of enterococcal infections (~80 %) are caused by *E. faecalis* due to its ability to acquire virulence traits and resistance to multiple antimicrobials (Gordon et al. 1992; Huycke et al. 1998; Ghoshal et al. 2006). The genome sequence of a clinical *E. faecalis* isolate V583 has revealed that more than a quarter of its genome consists of mobile or foreign DNA, including genes for vancomycin resistance and virulence markers (Paulsen et al. 2003). Vancomycin, once considered as the most powerful antimicrobial of “last resort,” is no longer efficacious in managing antimicrobial-resistant enterococci (Arias and Murray 2012). Further, the incidence of *E. faecalis* exhibiting antimicrobial-resistance viz. vancomycin resistance and virulence markers such as gelatinase (*gelE*), collagen binding protein (*ace*), endocarditis-associated antigen (*efaA*) and enterococcal surface protein (*esp*), have been explored mostly in clinical settings worldwide (Coque et al. 2002; Lowe et al. 1995; Eaton and Gasson 2001; Ghoshal et al. 2006; Mathur et al. 2003). However, very little is known about the distribution of antimicrobial-resistance and virulence-markers in *E. faecalis* isolates recovered from river waters (Novais et al. 2005; Moore et al. 2008; Lata et al. 2009b). River waters in developing countries are vulnerable to contamination due to discharge of untreated or partially treated municipal and industrial wastes, domestic and wild animal fecal waste and recreational activities on the banks (Ahmed et al. 2005; Ram et al. 2007). Such uninterrupted contamination of rivers lead to a bigger problem of nutrient enrichment or eutrophication of river waters. The eutrophication further leads to growth of aquatic macrophytes assisting the proliferation and prolonged survival of pathogens increasing public health risk (Whitman et al. 2003; Lata et al. 2009b).

**Fig. 1:**
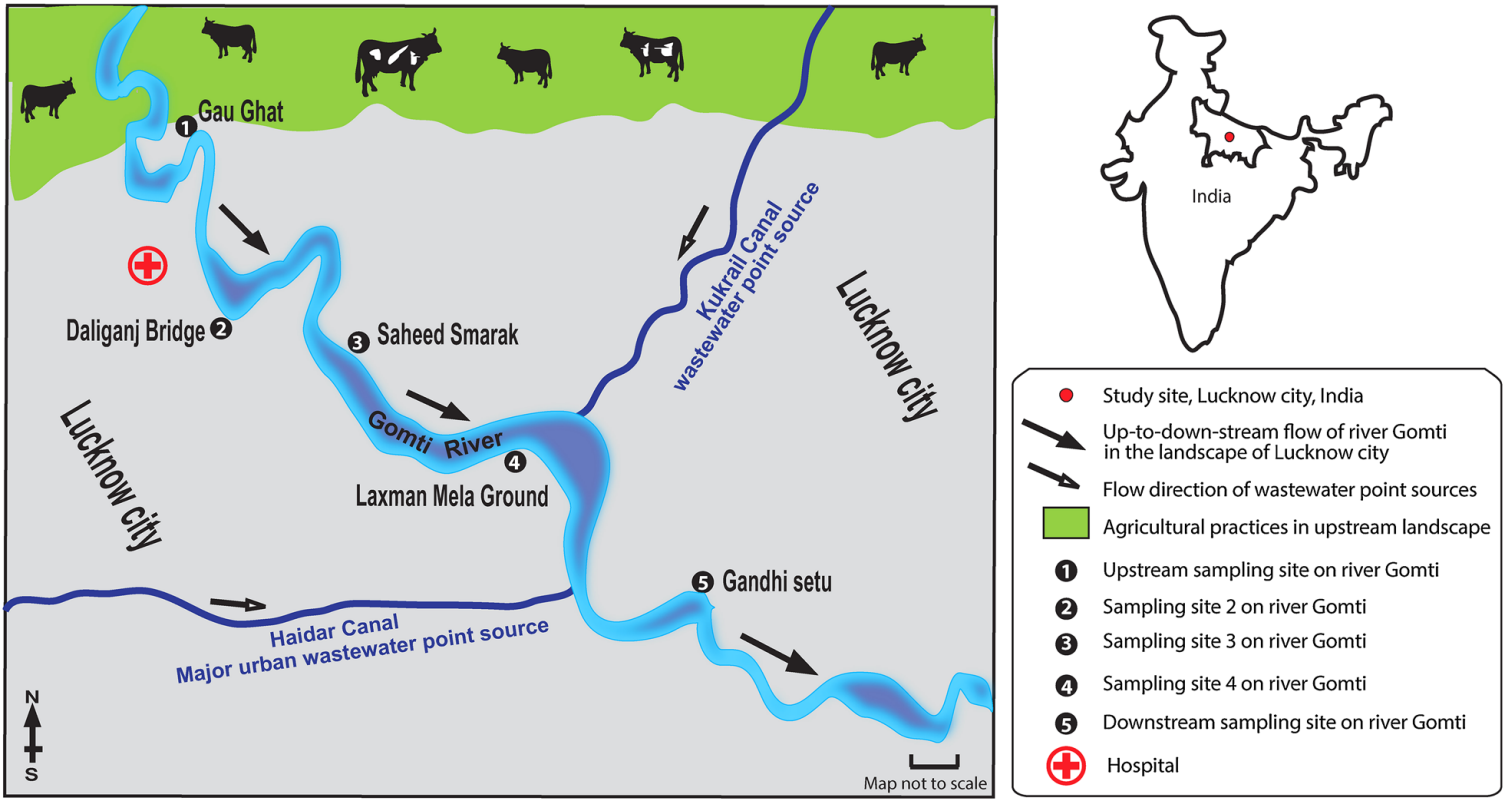
Map of study area/sampling sites in the landscape. Abbreviations: S#1, site 1: Gau-Ghat (the most upstream site), S#2, site 2: Daliganj Bridge, S#3, site 3: P Shaheed Smarak, S#4, site 4: Lakhshaman mela ground, S#5, site 5: Gandhi setu (the most downstream site). *Black*-*and*-*white arrows* indicate the direction of river water and wastewater flow in the up-to-downstream in the landscape respectively. Topographic data based upon Survey of the map of India Adopted from www.ttkmaps.com

River Gomti, a tributary of the river Ganga meets the major water requirement of more than 3.5 million populations of Lucknow city (Fig. 1). This river receives a discharge of 450 mLd untreated waste through different point sources due to the rapid industrialization and urbanization in the region (Singh et al. 2004). In India, river waters are used for drinking, bathing and carrying out various household chores. This demands determination of microbiological quality of river waters for remediation to protect public health. Reported prevalence of diarrheogenic and enterotoxigenic *E. coli* in the river waters of river Ganga and its tributary river Gomti has emphasized the necessity of information on recreational water quality ‘indicator’, *E. faecalis* in such riverine systems (Ram et al. 2007, 2008; Singh et al. 2010). This study investigates the prevalence and dissemination of vancomycin-resistant and pathogenic *E. faecalis* along the up-to-downstream landscape of Lucknow city (Fig. 1). The genotypic characterization is done by multiplex PCR assay panels comprising simultaneous amplification of four genes: *sodA*, *vanB*, *gelE* and either *ace* or *efaA*. In addition, the prevalence of *esp* virulence marker and multiple-antimicrobial-resistance was also examined amongst *E. faecalis* isolates from the river Gomti water.

## Results

### Concentration of enterococci

Median concentrations of enterococci increased gradually and significantly (p < 0.0001) in the river Gomti waters from up-to-down-stream sites in the landscape. The most downstream site was found to be 123 and > 4500-fold more polluted than the most upstream site, using MPN test and membrane filtration method, respectively (Table 1; Additional file 1: Table S1). Concentrations of enterococci at downstream sites were about 10, 23 and 69-fold higher at site 2, 3 and 4, respectively when compared to the most upstream site, using MPN test (Additional file 1: Table S1). At the same time, median concentration of enterococci using membrane filtration method was 45, 296, 512-fold higher at site 2, 3 and 4, respectively (Additional file 1: Table S1).

**Table 1:** Quantitative enumeration (log transformed) of enterococci collected from sites (n = 5) located on river Gomti in up-to-down-stream fashion

Sampling site	CFU/100 mL water [Median 95 % CI (lower–upper)]	MPN index/100 mLwater 95 % CI (lower–upper)^a^	*p* value^b^
Site 1	2.18 (2.16–2.21)	1.11 (0.70–1.58)	<0.0001***
Site 2	3.84 (3.83–3.84)	2.15 (1.78–2.56)	
Site 3	4.66 (4.65–4.67)	2.48 (2.00–3.11)	
Site 4	4.89 (4.89–4.90)	2.95 (2.48–3.46)	
Site 5	5.84 (5.84–5.84)	3.20 (2.78–3.72)	
Control^c^	ND	ND	

*ND* not detected

*** Statistically significant at alpha < 0.05

^a^Lower 95 % CI–upper 95 % CI is adopted from Table 9221.IV, Section 9221C. Estimation of bacterial density, APHA (1998); log transformed values shown in the Table 1; See Additional file 1: Table S1 for details

^b^ *p* value was calculated using Chi square test for trend, χ^2^ = 1636; df = 1

^c^Sterile Milli Q water used as control

### Multiplex PCR for vancomycin resistant pathogenic *E. faecalis*

**Fig. 2:**
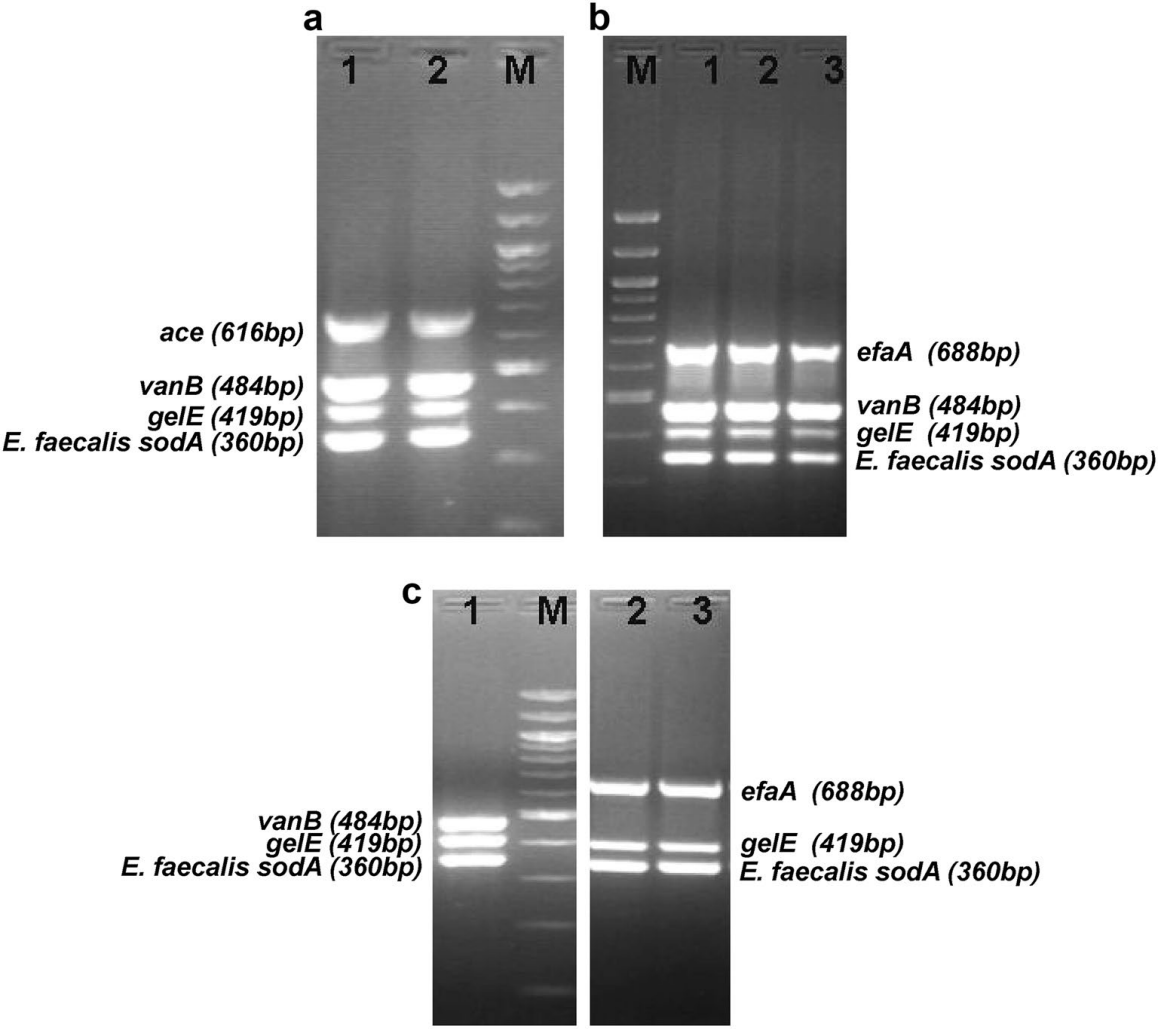
Multiplex PCR amplicons of *E. faecalis* isolates exhibiting *vanB* resistance and *gelE*, *ace*, *efaA* virulence-markers. **a** *sodA* ^+^ *gelE* ^+^ *vanB* ^+^ *ace* ^+^ genotype, *lane 1*: positive control (*E. faecalis* ATCC 51229), *lane 2*: 23E, *lane M*: 100 bp DNA ladder (New England Biolabs Inc.). **b** *sodA* ^+^ *gelE* ^+^ *vanB* ^+^ *efA* ^+^ genotype, *lane M* 100 bp DNA ladder (New England Biolabs Inc.), *lane 1* positive control (*E. faecalis* ATCC 51229), *lane 2* HE, *lane 3* GE and **c** *sodA* ^+^ *gelE* ^+^ *vanB* ^+^ genotype, *lane 1*: 20E, *lane M* 100 bp DNA ladder (New England Biolabs Inc.), *sodA* ^+^ *efaA* ^+^ *gelE* ^+^ genotype, *lane 2*: G3D, *lane 3* G3C

The multiplex PCR based genotypic characterization was carried out to determine the prevalence of *vanB* resistance and *gelE*, *ace* or *efaA* virulence markers *in E. faecalis* isolates from river Gomti (Tables 2 and 3; Fig. 2). The genotypic characterization has shown a significant distribution of virulence-markers along the landscape (p < 0.05). The median count investigated throughout the landscape was 3 (Table 4). According to genotypic characterization the frequency of *vanB* resistant *E. faecalis* is 27 % in the landscape. However, 100 % *E. faecalis* isolates recovered from river Gomti exhibit prevalence of *gelE* (gelatinase) and *esp* virulence markers. The *esp* gene was characterized as described earlier (Lata et al. 2009a). The *ace* gene was observed in ca. 58 % *E. faecalis* and the frequency of *efaA* virulence marker was ca. 97 % in the landscape. Further the frequency of various combinations of multiple-virulence-markers has been observed in the landscape (Table 3).

**Table 2:** Multiplex PCR primers for vancomycin-resistance and virulence-markers detection in river water isolates of *E. faecalis*

Multiplexed/singleplexed genes	Oligomer sequence (5′ → 3′)	Oligo-mer conc.^a^ (µM)	Product size (bp)	Reference
*sodA* (*E.faecalis*)	F: 5′-ACTTATGTGACTAACTTAACC-3′ R: 5′-TAATGGTGAATCTTGGTTTGG-3′	0.15	360	Jackson et al. (2004)
*vanB*	F: 5′-CAAAGCTCCGCAGCTTGCATG-3′ R: 5′-TGCATCCAAGCACCCGATATAC-3′	0.20	484	Dahl et al. (1999)
*gelE*	F: 5′-ACCCCGTATCATTGGTTT-3′ R: 5′-ACGCATTGCTTTTCCATC-3′	0.25	419	Eaton and Gasson (2001)
*ace* ^b^	F: 5′-GGAATGACCGAGAACGATGGC-3′ R: 5′-GCTTGATGTTGGCCTGCTTCCG-3′	0.29	616	Creti et al. (2004)
*efaA* ^b^	F: 5′-GCCAATTGGGACAGACCCTC-3′ R: 5′-CGCCTTCTGTTCCTTCTTTGGC-3′	0.30	688	Creti et al. (2004)
*esp* ^c^	F: 5′-TTGCTAATGCTAGTCCACGACC-3′ R: 5′-GCGTCAACACTTGCATTGCCGAA-3′	0.15	933	Eaton and Gasson (2001)

^a^Oligomer concentration optimized for this study

^b^Multiplexed genes include either *ace* or *efaA* gene with *sodA*, *vanB* and *gelE* genes

^c^Singleplexed gene

**Table 3:** Multiplex PCR based fingerprints of vancomycin-resistance and multiple-virulence-markers in *E. faecalis* isolates from river Gomti

*E. faecalis* isolates	Target genes					
	Housekeeping gene	Antimicrobial resistance and Virulence genes				
	*sodA* (*E. faecalis*)	*vanB*	*gelE*	*ace*	*efaA*	*Esp*
*E. faecalis* ATCC 51299 (ATCC, USA) (positive control)	+	+	+P	+	+	+
Site #1						
2E	+	−	+	−	+	+
AE	+	−	+P	−	+	+
BE	+	−	+P	+	+	+
FE	+	+	+	−	+	+
GE	+	+	+P	−	+	+
Site #2						
4E	+	+	+P	+	+	+
HE	+	+	+P	−	+	+
CE	+	+	+P	+	+	+
G2A	+	−	+P	−	+	+
G2B	+	−	+	+	+	+
Site #3						
9E	+	−	+P	−	+	+
IE	+	−	+	−	+	+
16E	+	−	+P	+	+	+
G3A	+	−	+P	+	+	+
G3B	+	−	+P	+	+	+
G3C	+	−	+	−	+	+
G3D	+	−	+	−	+	+
Site #4						
11E	+	−	+P	+	+	+
17E	+	−	+	+	+	+
18E	+	+	+P	+	+	+
19E	+	−	+P	−	+	+
20E	+	+	+P	−	−	+
G4A	+	−	+	+	+	+
G4B	+	−	+	+	+	+
Site #5						
15E	+	−	+P	−	+	+
21E	+	−	+	+	+	+
22E	+	+	+P	−	+	+
23E	+	+	+P	+	+	+
24E	+	−	+	+	+	+
G5A	+	−	+P	+	+	+
G5B	+	−	+P	+	+	+
G6A	+	−	+	+	+	+
G6B	+	−	+P	+	+	+

*ATCC* American Type Culture Collection, USA

+P denotes *E. faecalis* isolates exhibiting gelatinase phenotype

**Table 4:** Multiple-virulence-markers observed in each *E. faecalis* isolate from the up-to-down-stream landscape of Lucknow city

Sampling site	Virulence-markers characterized per isolate Median (range)	*p* value^a^
Site 1	3 (3–3)	0.0313*
Site 2	4 (3–4)	0.0313*
Site 3	3 (3–4)	0.0078*
Site 4	4 (1–4)	0.0078*
Site 5	4 (3–4)	0.0020*

^a^ *p* value was calculated using Wilcoxon rank sum test

* Statistically significant at alpha = 0.05

### Gelatinase exhibiting *E. faecalis* phenotypes

The phenotypic assay for determination of gelatinase activity shows high prevalence of gelatinase positive *E. faecalis* isolates in river Gomti throughout the landscape. Site 2 exhibits highest prevalence (80 %) of such isolates followed by the most downstream site 5 (67 %) and then most upstream site 1 (60 %) and 57 % for both site 3 and 4 (Table 3).

### Antimicrobial-resistance in *E. faecalis* isolates

A high frequency of multiple-antimicrobial-resistance was recorded among *E. faecalis* tested. The range for the number of antimicrobials against which resistance was observed in each *E. faecalis* isolates increased significantly (p < 0.05) towards downstream in the landscape (Tables 5, 6). The median of antimicrobial-resistance per isolate was nine at Gau-Ghat, the most upstream site and increased to a value of eleven at site 3 and subsequently declined to nine at site 4 and 5. In general, *E. faecalis* isolates were resistant to at least five antimicrobials throughout the landscape. However, the range of antimicrobial-resistance varied from 5 to 12 in the landscape (Table 6). The frequency of vancomycin-resistant *E. faecalis* (VRE; both VanA and VanB phenotypes) is very high throughout the landscape; at site 1, 60 % *E. faecalis* isolates are VRE and 40 % are VanB phenotype, at site 2: 100 % VRE and 60 % VanB, at site 3: 100 % VanA phenotype VRE, at site 4: 71 % VRE and 28.5 % VanB phenotype, at site 5: 88 % VRE and 22 % VanB phenotype of *E. faecalis* were observed (Table 5).

**Table 5:** Sitewise antimicrobial-resistance profile of *E. faecalis* isolates from River Gomti at Lucknow city

Sampling site	*E. faecalis* isolates	Antimicrobial resistance
Site #1	2E	Ox,T,G,E,M
	AE	A,T,P,G,E,Te,R,M,Va
	BE	A,S,Ox,T,G,P,E,R,M
	FE	Ox,T,G,R,M,Va
	GE	A,S,Ox,T,G,P,E,R,M,Va
Site #2	4E	A,Ox,P,G,E,R,Va,
	HE	S,Ox,T,K,G,E,R,M, Va
	CE	A,S,Ox,T,G,P,C,E,R,M,Va
	G2A	A,S,T,G,P,E,Te,R,M,Va
	G2B	A,S,Ox,T,G,P,E,Te,R,M,Va
Site #3	9E	A,T,G,P,Te,R,M,Va
	IE	A,S,Ox,T,P,C,G,E,Te,R,M,Va
	16E	A,S,Ox,T,G,P,E,Te,R,Va
	G3A	A,S,Ox,T,G,P,E,Te,R,M,Va
	G3B	A,S,Ox,T,P,G,E,Te,R,M,Va
	G3C	A,S,Ox,T,E, P,G,Te,R,M,Va
	G3D	A,S,Ox,T,G,P,Te,R,M,Va
Site #4	11E	S,G,E,R,M
	17E	A,S,OX,T,E,R,M
	18E	A,S,Ox,T,G,P,C,E,R,M,Va
	19E	A,S,Ox,T,P,C,E,Te,R,Va
	20E	S,T,G,P,E,R,M,Va
	G4A	A,S,Ox,T,G,P,Te,R,M,Va
	G4B	A,S,T,G,P,Te,R,M,Va
Site #5	15E	A,S,Ox,T,G,P,C,E,Te,R,M,Va
	21E	A,Ox,T,G,E,R
	22E	A,S,T,G,P,C,E,Va
	23E	A,S,Ox,G,P,C,E,Va
	24E	A,P,E,Te,R,M,Va
	G5A	A,S,Ox,T,G,P,E,Te,R,M,Va
	G5B	A,S,Ox,T,G,P,Te,R,M,Va
	G6A	A,S,Ox,T,G,P,Te,R,M,Va
	G6B	A,S,Ox,T,G,P,Te,R,M,Va

Antimicrobial abbreviation: *Nx* Norfloxacin, *A* ampicillin, *Ox* oxacillin, *P* penicillinG), *M* methicillin, *G* gentamycin, *S* streptomycin, *T* tetracycline, *C* chloramphenicol, *E* erythromycin, *R* rifampicin, *Va* vancomycin, *Te* teicoplanin

**Table 6:** Antimicrobial-resistance observed in each *E. faecalis* isolate in the up-to-down-stream landscape of Lucknow city

Sampling site	Antimicrobial-resistance per isolate Median (range)^a^	*p* value^b^
Site 1	9 (5–10)	0.0313*
Site 2	10 (7–11)	0.0313*
Site 3	11 (8–12)	0.0078*
Site 4	9 (5–11)	0.0078*
Site 5	9 (6–12)	0.0020*

^a^Median and range are reported to match more consistently with the nonparametric statistical tests performed

^b^ *p* value was calculated using Wilcoxon rank sum test

* Statistically significant at alpha = 0.05

## Discussion

This study was carried out to investigate the landscape dependent density and distribution of enterococci, prevalence of antimicrobial-resistance and virulence-markers among *E. faecalis* isolates recovered from river Gomti at Lucknow city. A multiplex PCR based genotypic characterization of vancomycin resistant pathogenic *E. faecalis* was carried out. The river Gomti exhibits high enterococci density at selected sites. The MPN index/100 mL values of enterococci recorded at all the sampling stations exceeded the standards set by regulatory authorities for river water reservoirs used for drinking and recreational purposes (US EPA 2003). The quantitative enumeration of enterococci by membrane filtration method has also shown a gradual increase in the concentration of enterococci towards the downstream sites in the landscape. These sites are highly polluted because of the recurring fecal pollution from nearby residential areas and cremation practices at local crematorium. Population pressure alongwith poor sanitation facilities might be responsible for the increased bacterial contamination in river waters of developing countries (Ram et al. 2007, 2008; Lata et al. 2009b). In the present study, an increasing trend of enterococci concentration was observed in the range of 1.5–1.6 × 10^2^ CFU/100 mL, 6.8–6.9 × 10^3^ CFU/100 mL, 4.4–4.6 × 10^4^ CFU/100 mL, 7.7–7.8 × 10^4^ CFU/100 mL and 6.8–6.9 × 10^5^ CFU/100 mL at sites 1, 2, 3, 4 and 5 respectively. The most downstream site in the landscape, Gandhi setu receives contamination from city through two major wastewater point sources, including Kukrail nala and city’s largest crematorium located just upstream. Internationally, the single-sample advisory limit of enterococci for fresh water is 61 CFU/100 mL, and the 5-day geometric mean should not exceed 33 CFU/100 mL, while Indian standards have not defined the limit for enterococci in terms of CFU/100 mL (CPCB 2002; USEPA 2003).

**Fig. 3:**
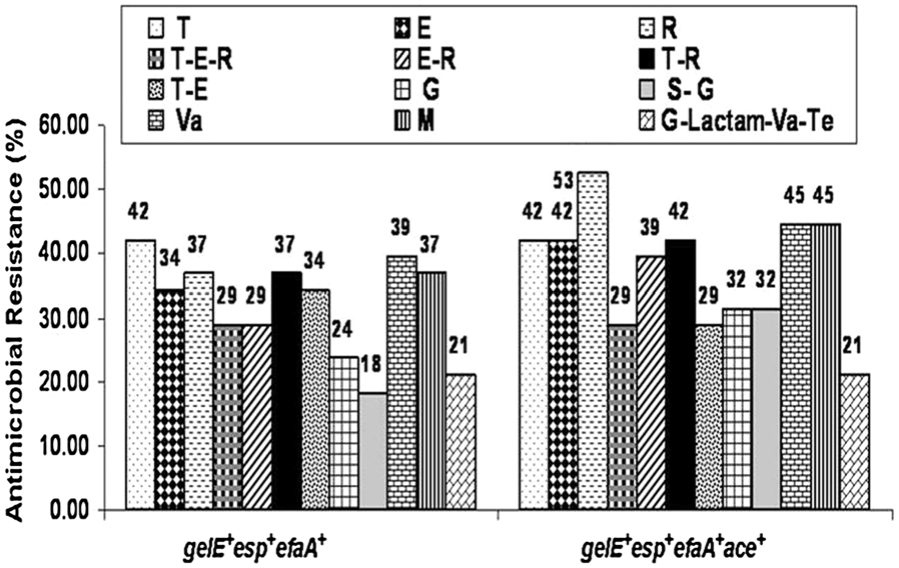
Frequency of various combinations of multiple-antimicrobial-resistance and multiple-virulence-markers among *E. faecalis* in the landscape. Abbreviations: T tetracycline, E erythromycin, R rifampicin, T–E–R tetracycline–macrolide–rifamycin, E–R (macrolide–rifamycin), T–R (tetracycline–rifamycin), T–E, (tetracycline–macrolide), G, gentamicin, S–G, streptomycin–gentamicin (aminoglycoside), Va, vancomycin (glycopeptide), M, methicillin (β-Lactam), G-Lactam-Va-Te (gentamicin-β-Lactam*-vancomycin-teicoplanin), *A–P–Ox–M ampicillin–penicillinG–oxacillin–methicillin (β-Lactam). All antimicrobial combinations derived from aforementioned antimicrobial abbreviations

A panel of multiplex PCR assay was developed in the current study for detection of vancomycin-resistant pathogenic *E. faecalis* in environmental waters (Table 2; Fig. 2). The frequency of *E. faecalis* isolates exhibiting multiple-virulence-markers and *vanB* genotype is 27 % in the landscape. However, 100 % *E. faecalis* isolates recovered from river Gomti exhibit prevalence of *gelE* and *esp* virulence markers. Gelatinase, a virulence marker widely reported in enterococci, hydrolyzes a broad range of bioactive substances and has been implicated in various infection models through biofilm formation (Parsek and Singh 2003; Nallapareddy et al. 2006). A recent study has shown the prevalence of gelatinase phenotype of enterococci with biofilm formation potential in agricultural environment and implicated it as a reservoir of clinically relevant strains (Macovei et al. 2009). The most frequent multiple-virulence-markers were *gelE*^+^*esp*^+^*efaA*^+^ and *gelE*^+^*esp*^+^*efaA*^+^*ace*^+^. Further the frequency of various combinations of multiple-antimicrobial-resistance and multiple-virulence-markers has been observed in the landscape. Among all, 39 % isolates were characterized with *gelE*^+^*esp*^+^*efaA*^+^ virulence markers and vancomycin resistance phenotype whereas 45 % were having methicillin resistance, VRE phenotype and *gelE*^+^*esp*^+^*efaA*^+^*ace*^+^ multiple-virulence-markers. Additionally 21 % of *gelE*^+^*esp*^+^*efaA*^+^*ace*^+^ isolates were observed to exhibit resistance to aminoglycoside–β-lactam–vancomycin–teicoplanin (G–Lactam–Va–Te); see Fig. 3 for details. The high frequency of VRE in the landscape, its association with other widely disseminated antimicrobials and virulence markers may lead to evolution of potential pathogenic VRE (Cetinkaya et al. 2000; Arias and Murray 2012). Additionally, 29 % isolates exhibit resistance to a macrolide, erythromycin, and rifampicin in association with tetracycline. A study from Denmark has reported carriage of coupled resistance of copper and antimicrobials (macrolide and glycopeptide) in pig-borne enterococci isolates contemplating persistence of the background pool of antimicrobial-resistance (BPAR) in that geographic region (Hasman and Aarestrup 2005). A set of other studies have reported the phenomenon of sustained BPAR in poultry and local population and its environmental carriage by plasmid maintenance systems (Sletvold et al. 2007b; Garcia-Migura et al. 2008). Moreover, previous study has implicated the possible transfer of linked virulence-traits and antimicrobial-resistance viz. vancomycin resistance (Heaton et al. 1996). The persistence of VRE in the environment even in the absence of antimicrobial selection pressure has been attributed to multiple types of plasmid maintenance systems that might play a pivotal role in persistence and dissemination of dangerous antimicrobial-resistant enterococci superbugs. (Pandey and Gerdes 2005; Sletvold et al. 2007a; Garcia-Migura et al. 2008). In general, *E. faecalis* isolates from the river Gomti exhibited resistance to 5–12 antimicrobials. The highest levels of multiple-antimicrobial-resistant *E. faecalis* were isolated from site 3 and 5 followed by sites 2, 4 and 1. Prevalence of such a high background pool of multiple-antimicrobial-resistance may be due to the lack of dilution of the contamination that is added to the river via various point and non-point sources. The river Gomti exhibits sluggish flow except for the monsoon period in the landscape. Although the specific sources of contaminants were not definitively determined, it is likely that the contaminants from nearby slums, cattle herds, poultries and the hospitals contributed to this contamination. Twenty-five city drains in the Lucknow area drain into the river Gomti (CPCB 2005). Heavy silting of the river reduces its carrying capacity. However, agricultural practices, poultry and dairy farming along site 1, the most upstream site might be a contributory factor for high prevalence of multiple-antimicrobial-resistance in the *E. faecalis* isolates recovered from this site. Although there is no data available from India in this context, the feed usage patterns in dairy farming or livestock operations and manure application in the agricultural practices are reported to be important contributing factors elsewhere (Aarestrup et al. 2000; Thurston-Enriquez et al. 2005). The VRE, both VanA and VanB phenotypes were ubiquitously present throughout the landscape. The increased frequency (100 %) of VRE at site 2 may be due to addition of contamination from the hospital located just upstream.

The river Gomti identified as one of the most polluted rivers in India, is the lifeline of Lucknow city (population size ~3.5 million) as it meets about half of the total domestic water demand (about 550 mLd) of the city (Singh et al. 2004). In India, large population depends on untreated river water for carrying out daily needs (Hamner et al. 2006; Ram et al. 2007). The prevalence of vancomycin-resistant pathogenic *E. faecalis* isolates in river Gomti is a threat to public health as superbugs like VRE are among the most critical human health challenges of this century (Pruden et al. 2006). This study reports vancomycin-resistant pathogenic *E. faecalis* in river Gomti waters along an up to downstream city landscape. The emerging VRE and high background pool of multiple-antimicrobial-resistant and multi-virulent *E. faecalis* can contribute to the dissemination of resistance and virulence-markers in the diverse enterococci and other bacteria. Therefore, the presence of antimicrobial-resistant pathogenic *E. faecalis* in river waters of developing nations like India demands immediate environmental intelligence and surveillance for risk assessment and prevention strategies for protection of public health.

## Conclusion

The occurrence of pathogenic VRE in river Gomti surface water is an important health concern. The observed high background pool of antimicrobial resistance and virulence in *E. faecalis* in river waters has the potential to disseminate more alarming antimicrobial resistance in the environment and poses serious health risk in developing countries like India as VRE gut inhabitation could lead to increased economic burden for healthcare.

## Methods

### Study site and Sample collection

The study was conducted along 10 km stretch of river Gomti in Lucknow city (Latitude: 25.55′N, Longitude: 80.58′E and Altitude: 123 m). River Gomti, flowing through the northern part of the country, is a major tributary of the Ganga River in India. Originating from a natural reservoir in the swampy and densely forested area near Madho-Tanda (altitude 200 m; latitude 28°34′N and E longitude 80°07′E) in the foothills of Himalayas, the river traverses a distance of about 730 km in the Indo-Gangetic alluvial region before its confluence with river Ganga. The river flowing through the Lucknow city (population: ~3.5 million), the State capital of Uttar Pradesh, meets about half of the total domestic water demand (about 550 mLd) of the town. The average flow of the river at Lucknow varies between 500 mLd in summers and 55,000 mLd during the monsoons. The river contains water throughout the year and exhibits sluggish flow except for the monsoon period. The Gomti River, identified as one of the most polluted tributary of river Ganga, receives 450 mLd of untreated domestic waste water in Lucknow city (Singh et al. 2004). In this study, five sites located in up-to-downstream on the river, namely Gau-ghat (site 1: located in upstream of river), Daliganj Bridge (site 2: bathing ghat and holy spot), Shaheed Smarak (site 3: bathing ghat and picnic spot), Lakshaman mela ground (site 4: recreational and picnic spots), Gandhi setu (site 5, most downstream location in the landscape) were selected based on various human activities on the banks of river Gomti. A cross-sectional approach was used to collect river water samples. Samples were collected in triplicate (n = 15) from five locations situated in up-to-down-stream fashion (Fig. 1). In brief, three transects were established randomly at each site and water samples (1 L) were collected 30 cm below the water surface from left, mid and right banks of the river along each transect. The samples of river water were stored in sterile glass bottles, labeled and transported on ice to the laboratory for analysis. Sample processing and analysis were conducted within 6 h after sample collection.

### Isolation and enumeration of Enterococci

Quantitative enumeration of fecal streptococci or enterococci at selected sites was performed using the standard multiple tube fermentation technique (APHA 1998). In brief, tubes containing 20 mL Azide Dextrose Broth (ADB) were prepared. A series of tubes of ADB was inoculated with appropriate graduated quantities of sample (10, 1 and 0.1 mL). The inoculated tubes were incubated at 37 ± 0.5 °C and examined for turbidity after 24–48 h and calculated MPN index/100 mL surface water depending upon the number of positive tubes. Enterococci were recovered from each sample using standard membrane filtration method (APHA 1998). Briefly, tenfold dilutions of each water sample was prepared (10^0^, 10^−1^, 10^−2^ and 10^−3^), and 10 mL of each dilution were filtered through 0.45-µm, 47-mm mixed cellulose ester filters (Millipore, Bedford, MA, USA) The filters were then placed on mE agar plates incubated at 41 ± 0.5 °C for 48 h for detection and enumeration of enterococci (Hi-Media, India). After 48 h, membrane filters from mE agar plates were placed on Bile Esculin Azide (BEA) agar plates and incubated at 41 ± 0.5 °C for 20 min. Colonies characteristic of enterococci, ranging from pink to dark red on mE agar and producing a brown to black precipitate on BEA agar, were considered presumptive enterococci (APHA 1998). Negative control filters and agar plates were included in each membrane filtration analysis. All colonies were counted, and concentration of enterococci per 100 mL water was determined from dilution plates containing 30–300 CFU. Presumptive enterococci recovered (n = 60) from each sample were identified by biochemical tests, including catalase test and PYR test. The growth of isolates was determined in 6.5 % NaCl, pH 9.6, and at 10°s and 45 °C, respectively. All confirmed enterococci isolates were archived in tryptic soy broth with 15 % (v/v) glycerol at -80 °C for further analyses.

### Multiplex PCR assay

All enterococci isolates confirmed by biochemical tests were subjected to genotypic characterization by multiplex Polymerase Chain Reaction (PCR) technique. The multiplex PCR assay format was designed comprising four genes; either ace or *efaA* gene with *sodA* (specific for detection of *E. faecalis*), *vanB* and *gelE* gene. All the primers used in this assay have been reported earlier in singleplex PCR format for detection of respective genes in clinical settings (Table 2; supplementary information). Genomic DNA was extracted from overnight grown cells of enterococci isolates (n = 60) using Genelute bacterial genomic DNA kit (Sigma, USA). PCR assay was performed in reaction volume of 50 µl. In brief, The mastermix consisted of 1× hot start Taq buffer, 3.0 mM MgCl_2_, 0.4 mM deoxynucleotide triphosphate mix, 1.5 U of Hotstart Taq polymerase (MBI Fermentas), 300 pg of template DNA, sterile molecular grade water, forward and reverse primer (Metabion, Germany) concentration optimized for this study has been described in (Table 2; supplementary information). The PCR protocol employed an initial denaturation at 95 °C for 3 min followed by 30 cycles of denaturation at 95 °C; 30 s, annealing at 51 °C; 30 s, elongation at 72 °C; 50 s and final extension at 72 °C for 8 min. The amplicons were electrophoresed on 1.5 % agarose gel in Tris–acetate-EDTA buffer supplemented with 0.5 µg/mL of ethidium bromide. Standard DNA molecular weight markers used were 100 bp DNA ladder (MBI Fermentas, USA). The presence of gene *esp* encoding enterococcal surface protein in different *E. faecalis* isolates was also examined (Table 5.2). *E. faecalis* ATCC 51229, *E. faecium* ATCC 35667, 27270, *E. durans* ATCC 49470, *E. hirae* ATCC 9790, *E. coli* MTCC 723*, Vibrio cholerae* ATCC 51394, *Salmonella typhimurium* ATCC 13311 and *Salmonella typhimurium* ATCC 14028 were used throughout the study as reference/standard strains.

### Gelatinase phenotype determination

Gelatinase production was detected by spotting mid-log phase grown pure culture of individual *E. faecalis* isolates (n = 33) on freshly prepared tryptic soy agar plates (Himedia, India) containing 1.5 % of skimmed milk. Plates were incubated overnight at 37 °C and then cooled to ambient temperature for 2 h. The appearance of a transparent halo around the colonies was considered to be a positive indication of gelatinase production (Gilmore et al. 2002).

### Antimicrobial susceptibility testing

A panel of thirteen antimicrobials (antimicrobial abbreviation:mcg/disc) impregnated on paper discs (Himedia Ltd., India) belonging to eight different group of antimicrobials as Fluoroquinolone: Norfloxacin (Nx:10mcg), β-lactam: Ampicillin (A:10mcg), Oxacillin (Ox:1mcg), PenicillinG (P:10 units), Methicillin (M:5mcg), Aminoglycoside: Gentamycin (G:10mcg), Streptomycin (S:10mcg), Tetracycline: Tetracycline (T:30mcg), Phenicol: Chloramphenicol (C:30mcg), Macrolide: Erythromycin (E:15mcg), Rifamycin: Rifampicin (R:5mcg), Glycopeptides: Vancomycin (Va:30mcg), Teicoplanin (Te:30mcg) were used for testing the sensitivity of isolated organisms by Kirby-Bauer disc diffusion method as described by Clinical Laboratory Standards Institute (CLSI 2002). In brief, pure *E. faecalis* isolate culture colonies (3–4) were transferred to tubes containing 5 mL brain heart infusion broth and incubated in rotary shaker (Innova 4230, New Brunswick, USA) at 150 rpm and 37 + 1 °C for 4–6 h to yield a suspension of 10^6^ cells/mL. The inoculum was uniformally spread on the sterile Muller-Hinton agar plates (90 mm diameter) using the sterile cotton swab. Four to six antimicrobial discs were applied aseptically, at least 24 mm apart on Mueller–Hinton agar plates. The plates were incubated immediately at 35 + 1 °C; 16–18 h for all enlisted antimicrobials and 24 h for vancomycin. The test was performed in triplicate for each *E. faecalis* isolate. The diameter of zones showing inhibitions were measured to the nearest mm and recorded. A zone size interpretive chart was used to determine sensitivity/resistance of antimicrobials (CLSI 2002).

### Determination of Vancomycin-resistant VanA and VanB phenotype of *E. faecalis*

#### VanA phenotyping

Antimicrobials: Vancomycin (Van) and Teicoplanin (Te) were used for this study (Sigma-aldrich, USA). After Biochemical and microbiological confirmation, *E.faecalis* isolates from each sampling site were subjected to tube macrodilution method of antimicrobial susceptibility testing (Van: ≥ 64 µg/mlL, Te: ≥ 16 µg/mL) with slight modification. In brief, well characterized *E. faecalis* isolates were grown in tubes containing 5 mL Brain Heart Infusion broth and incubated in rotary shaker (INNOVA 4230, New Brunswick, USA) at 150 rpm and 37 + 1 °C for 4–6 h to yield a suspension of 10^6^ cells/mL. The inoculum of 10^6^ cells was added to sterile Muller-Hinton broth tubes containing Van: ≥ 64 µg/mL, Te: ≥ 16 µg/mL. The tubes were incubated immediately at 35 + 1 °C for 24 h. The tubes showing consistent growth were recorded as VanA positive. E. faecium ATCC 51559 was used as VanA positive and sterile water as negative control.

#### VanB phenotyping

Antimicrobial Vancomycin (Van) was used for this study (Sigma-aldrich, USA). The *E. faecalis* isolates from each sampling site were subjected to tube macrodilution method of antimicrobial susceptibility testing (Van: 4–1024 µg/mL) with slight modification. The tube macrodilution method CLSI (2005) for antimicrobial susceptibility testing (Van: 4–1024 µg/mL) was adopted with slight modification. In brief, well characterized *E. faecalis* isolates were grown in tubes containing 5 mL Brain Heart Infusion broth and incubated in rotary shaker (INNOVA 4230, New Brunswick, USA) at 150 rpm and 37 + 1 °C for 4–6 h to yield a suspension of 10^6^ cells/mL. The inoculum of 10^6^ cells was added to sterile Muller-Hinton broth tubes containing Van: 4–1024 µg/mL in twofold dilution series. The tubes were incubated immediately at 35 + 1 °C for 24 h. The tubes showing consistent growth were recorded as VanB positive. *E. faecalis* ATCC 51299 served as VanB positive and sterile water as negative control.

### Statistical analyses

The concentrations of enterococci obtained using MPN analysis test and membrane filtration method from up-to-downstream river water samples was compared, Chi square test for trend was applied for the purpose. The prevalence and distribution of antimicrobial-resistance and virulence-markers among isolates from up-to-downstream landscape was assessed using wilcoxon rank-sum tests. All statistical tests were performed using GraphPad Prism version 6.0 for Windows (GraphPad Software, San Diego, CA, USA, www.graphpad.com).

## Additional file

10.1186/s40064-016-2870-5 Quantitative enumeration of enterococci collected from sites (n=5) located on river Gomti in up-to-down-stream fashion (See Table 1 for log transformed values).

## Abbreviations

VRE: vancomycin resistant enterococci
PCR: polymerase chain reaction
BPAR: background pool of antimicrobial resistance
MPN: maximum probable number
CFU: colony forming unit
