# Supplementary material for: Multiplex PCR based genotypic characterization of pathogenic vancomycin resistant Enterococcus faecalis recovered from an Indian river along a city landscape
Source: Springerplus. 2016 Jul 28;5(1):1199. doi: 10.1186/s40064-016-2870-5 (PMC4963349; doi:10.1186/s40064-016-2870-5)
Supplement: Supplementary file 1 — 10.1186/s40064-016-2870-5 Quantitative enumeration of enterococci collected from sites (n=5) located on river Gomti in up-to-down-stream fashion (See Table 1 for log transformed values). [file 40064_2016_2870_MOESM1_ESM.docx]

**Additional file 1: Table S1.** Quantitative enumeration of enterococci collected from sites (n=5) located on river Gomti in up-to-down-stream fashion

| Sampling Site | CFU/100 mLwater  [Median 95% CI  (Lower - Upper)] | MPN index/100 mLwater  95% CI (Lower - Upper)^a^ | *p*-Value^b^ |
| --- | --- | --- | --- |
| Site 1 | 153 (145 - 162) | 13 (5 - 38) | <0.0001*** |
| Site 2 | 6900 (6839 - 6991) | 140 (60 - 360) |  |
| Site 3 | 45300 (44373 - 46354) | 300 (100 - 1300) |  |
| Site 4 | 78430 (77660 - 78930) | 900 (300 - 2900) |  |
| Site 5 | 692000 (686082 - 698585) | 1600 (600 - 5300) |  |
| Control^c^ | ND | ND |  |

^a^Lower 95% CI - Upper 95% CI are adopted from Table 9221.IV, Section 9221C. Estimation of Bacterial density, APHA (1998). ^b^*p*-Value was calculated using chi square test for trend, χ^2^ = 1636; df = 1. ^c^Sterile Milli Q water used as control. ***Statistically significant at alpha <0.05. Abbreviations: ND, Not Detected.
